# Frecuencia y caracterización de la automedicación ante manifestaciones dentales en pacientes que acudieron a clínicas privadas en Lima en el año 2021

**DOI:** 10.21142/2523-2754-1001-2022-097

**Published:** 2022-03-30

**Authors:** Aristides Arriarán Cisneros, Melissa Becerra Bravo, Eliberto Ruiz Ramirez

**Affiliations:** 1 Carrera de Estomatología, Universidad Científica del Sur. Lima, Perú. aristidesarriaran@gmail.com, melissa.becerra@unmsm.edu.pe Universidad Científica del Sur Carrera de Estomatología Universidad Científica del Sur Lima Peru aristidesarriaran@gmail.com melissa.becerra@unmsm.edu.pe; 2 Programa de Maestría en Farmacología. Universidad Nacional Mayor de San Marcos, Lima, Perú. eruizr@cientifica.edu.pe Universidad Nacional Mayor de San Marcos Programa de Maestría en Farmacología Universidad Nacional Mayor de San Marcos Lima Peru eruizr@cientifica.edu.pe

**Keywords:** automedicación, dental, dolor, self-medication, dental, pain

## Abstract

**Introducción:**

La automedicación es la práctica de usar fármacos que no han sido recetados por un profesional de la salud, lo que muchas veces, en vez de ayudar, agrava la salud del paciente. El dolor dental es el principal motivo para la realización de este acto en el área de la odontología. Es importante conocer si los pacientes se automedican para desarrollar estrategias para reducir el mal uso de medicamentos y evitar esta práctica.

**Objetivo:**

Determinar la frecuencia y la caracterización de la automedicación en los pacientes que acudan a las tres clínicas odontológicas privadas entre los meses de marzo a julio del 2021.

**Materiales y métodos:**

Estudio observacional de tipo transversal cuya muestra fue de 450 pacientes de tres clínicas odontológicas de la ciudad de Lima. El instrumento de evaluación fue un cuestionario de 13 preguntas sobre las principales características de la automedicación.

**Resultados:**

El 85,8% de los pacientes consumen medicamentos sin prescripción médica. El 41,5% de los pacientes se automedicaron por prescripciones antiguas realizadas por un odontólogo. El grupo etario que más se automedicó fue el de 26 a 35 años, con un 35,5%. El tipo de dolencia más frecuente fue el dolor dental, con un 45,6%. El grado de instrucción con mayor porcentaje fue el superior universitario completo, con un 32,9%.

**Conclusiones:**

La frecuencia de automedicación de los pacientes que acudieron a los tres centros dentales es considerablemente alta.

## Abstract (en)

**Introduction:**

The self-medication is the use of drugs without health professionals’ prescription that many times, instead of helping, aggravate the patient's health. Dental pain is the main reason for performing this act in the area of odontology. It is important to know if patients self-medicate in order to develop strategies to reduce the misuse of drugs and avoid this practice.

**Objective:**

Establish the frequency and characterization of self-medication in patients who visit to the three private dental clinics between the months of march to July in the year 2021.

**Materials and methods:**

Cross-sectional observational study with a sample of 450 patients from three dental clinics in the city of Lima. The assessment tool was a 13-question quiz about the main characteristics of self-medication.

**Results:**

The 85,8% of patients use drugs without medical prescription. 41.5% of the patients self-medicated did it with old prescriptions made by the dentist. The age group that more self-medicated was 26-35 years old with 35.5%. The most frequent type of disease was dental pain with 45.6%, The degree of instruction with the highest percentage was complete university superior with 32.9%.

**Conclusions:**

The frequency of self-medication of the patients who visited the three dental centers is considerably high.

## INTRODUCCIÓN

La Organización Mundial de la Salud (OMS) define la automedicación como “el uso de medicamentos por decisión propia o por consejo de otra persona que no tiene conocimientos sobre los medicamentos o sobre la enfermedad” ^1^^,^^2^. En la actualidad, la práctica de la automedicación se encuentra muy difundida en los países desarrollados y en vías de desarrollo ^1^, mientras que la población joven y adulta joven suele ser la que más realiza dicha práctica ^3^.

La automedicación es una práctica muy habitual y que se encuentra en incremento, la cual es llevada a cabo por razones sociales, económicas y culturales que pueden generar consecuencias severas para la salud, al promover la administración de medicamentos sin tener en cuenta un diagnóstico preciso de la enfermedad que se padece ^4^. Entre los factores que contribuyen a que esta práctica se dé muy frecuentemente en la población están la amplia variedad de medicamentos de venta libre, la poca regulación que existe en el control de medicamentos por las farmacias, y las campañas publicitarias que resaltan y exageran los efectos benéficos de los fármacos, sin dar a conocer a los consumidores sus efectos adversos ^5^. 

En los últimos tiempos, el colapso de los sistemas de salud a causa de las emergencias sanitarias ha provocado la carencia de citas para los servicios de salud en el corto plazo, largas esperas para acceder a los tratamientos médicos y mucha inconformidad por parte de los pacientes. En consecuencia, algunas prácticas, como la automedicación, basada en recetas de otros pacientes o en recomendaciones de externos, se hace más frecuente ^6^.

Entre las principales afecciones que llevan a la población a automedicarse se encuentra el dolor ^7^, especialmente en el caso de problemas odontológicos ^8^^,^^9^. El dolor dental es el motivo de consulta más frecuente en odontología, lo que lleva al uso de diversos tipos de analgésicos, opiáceos y no opiáceos, para reducir el dolor percibido, y muchos de estos medicamentos son utilizados inadecuadamente por los pacientes sin tener una correcta indicación médica-odontológica ^10^^,^^11^. 

Por lo expuesto, la siguiente investigación tiene como objetivo determinar la frecuencia y caracterización de la automedicación en pacientes que acuden a servicios de salud privados en la ciudad de Lima, Perú, en el año 2021. 

## MATERIALES Y MÉTODOS

Se realizó un estudio observacional de tipo transversal en 450 pacientes mayores de 18 años de tres clínicas odontológicas de la ciudad de Lima, entre los meses de marzo y julio del 2021. Para llevar a cabo el estudio, se contó la aprobación del Comité Institucional de Ética de la Universidad Científica de Sur (Lima, Perú), con código de resolución de aceptación 306-2020-PRE99.

Se utilizó un cuestionario validado por Conhi ^4^ y que estuvo constituido de dos partes. La primera parte consistió en preguntas sobre las características personales del paciente como la edad, el sexo, el grado de instrucción y la ocupación. La segunda parte incluyó preguntas relacionadas con datos para evaluar la prevalencia de la automedicación y las características de los fármacos consumidos.

Se solicitó el permiso de las autoridades correspondientes de las tres clínicas dentales para la entrega de los cuestionarios a los pacientes que ingresen a sus establecimientos. Una vez entregadas las encuestas a los pacientes que aceptaron voluntariamente participar de este estudio, se les informó, en un tiempo no mayor a 5 minutos, en qué consistía la investigación y que los resultados no serían publicados individualmente. Luego de brindarles esta información, se les entregó el consentimiento informado con el cuestionario impreso y se les otorgó un tiempo de 20 minutos para responder las preguntas.

Los datos obtenidos fueron ingresados en una hoja de Excel de Microsoft y el análisis se realizó con el programa IBM SPSS (versión 21.0). En esta investigación, al tener variables de forma cualitativas, se utilizó la prueba de chi cuadrado. Asimismo, se trabajó con un nivel de significancia de 0,05. Los parámetros descriptivos se analizaron y representaron en números, tablas y gráficos.

## RESULTADOS

**Figura 1:**
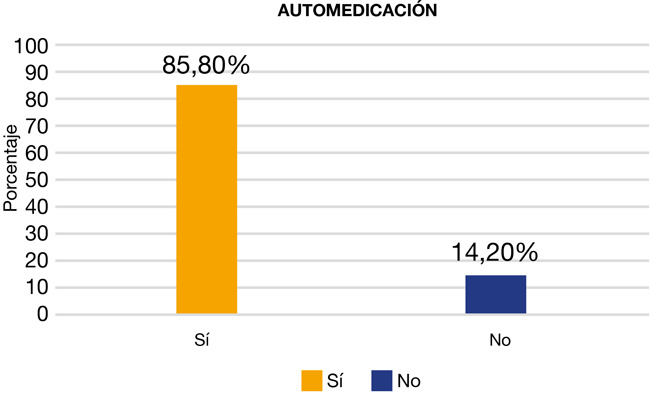
Frecuencia y caracterización en pacientes que acudieron a tres clínicas odontológicas privadas entre los meses de marzo y julio del 2021

La muestra estuvo constituida por 450 pacientes, de los cuales el 85,8% marcó la opción que decía que sí se automedicaba y el 14,5% señaló que no realizaba dicho acto (Figura 1). 

Del total de la muestra (n = 450), el 51,1% fueron del género femenino y el 48,9%, del género masculino. Respecto del grupo de edades, predominó el de 26 a 35 años, con un 34,8%. Según el grado de instrucción, el 33,3% indicó que tenía educación superior universitaria y, de acuerdo con su ocupación, el 41,1% eran profesionales. En tanto, el 85,5% señaló que se automedicaba y el 14,5% indicó que no realizaba dicho acto.

Con relación a la frecuencia y la caracterización de la automedicación según la edad, se observó que los pacientes de 26 a 35 años fueron los que más se automedicaban (35,5%), por lo que se determinó la existencia de una diferencia estadísticamente significativa con las demás alternativas (p = 0,036) (Tabla 1). De acuerdo con el género, se dio un predominio en el sexo femenino (50,3%) en comparación con el género masculino (49,7%); esta diferencia no fue estadísticamente significativa (p = 0,153). 

**Tabla1.** Frecuencia y caracterización de automedicación según sexo, edad, ocupación y grado de instrucción en los pacientes que acudieron a tres clínicas odontológicas privadas de Lima en el año 2021

Variables	Automedicación				p
	Sí	%	N.^o^	%	
Edad					0,036
18-25	136	35,2	14	21,9	
26-35	137	35,5	20	31,3	
36-45	68	17,6	15	23,4	
46-55	21	5,4	6	9,4	
>55	24	6,2	9	14,1	
Total	386	100	64	100	
Sexo					0,153
Masculino	192	49,7	38	59,4	
Femenino	194	50,3	26	40,6	
Total	386	100	64	100	
Ocupación					0,139
Ama de casa	16	4,1	2	3,1	
Estudiante	102	26,4	9	14,1	
Desocupado	8	2,1	0	0	
Trabajador independiente	105	27,2	23	35,9	
Profesional	155	40,2	30	46,9	
Total	386	100	64	100	
Grado de instrucción					0,778
Analfabeto	1	0,3	0	0	
Primaria incompleta	2	0,5	0	0	
Primaria completa	2	0,5	1	1,6	
Secundaria incompleta	4	1,0	1	1,6	
Secundaria completa	36	9,3	9	14,1	
Superior técnica incompleta	52	13,5	8	12,5	
Superior técnica completa	68	17,6	12	18,8	
Superior universitaria incompleta	94	24,4	10	15,6	
Superior universitaria completa	127	32,9	23	35,9	
Total	386	100	64	100	

Respecto de la ocupación, los que presentaron mayor frecuencia de automedicación fueron los profesionales (40,2%), en comparación con los demás grupos, diferencia que no fue estadísticamente significativa (p = 0,139). El grado de instrucción con mayor frecuencia de automedicación fue el superior universitario completo (32,9%) en relación con los demás grupos; esto no representó una diferencia estadísticamente significativa (p = 0,778) (Tabla 1).

Se encontró que la mayor prevalencia de la automedicación se debía al dolor dental, con un 45,6% (n = 176), y en menor cantidad a los traumatismos, con un 1,6% (n = 6) (Tabla 2).

**Tabla 2:** Frecuencia y caracterización de automedicación según el tipo de dolencia en los pacientes que acudieron a tres clínicas odontológicas privadas de Lima en el año 2021

Tipo de dolencia	General	
	N.^o^	%
Dolor dental	176	45,6
Inflamación	100	25,9
Infección	28	7,3
Traumatismos	6	1.6
Otros	76	19,7
Total	386	100

Con relación a la frecuencia y la caracterización de la automedicación según recomendación, se observó que el 41,5% (n = 160) recibió la recomendación de un odontólogo, el 14,2% (n = 55) la recibió de un farmacéutico y el 4,9% (n = 19), por parte de sus amistades. Según el motivo de la medicación, predominó “por hábitos”, con el 23,8% (n = 92), mientras que el 16,3% (n = 63) indicó “porque es muy cara la consulta”. Respecto del lugar donde adquirió el medicamento, el 78% (n = 301) de pacientes indicó “en la farmacia” (Tabla 3).

**Tabla 3:** Frecuencia y caracterización de automedicación según recomendación, motivo y lugar donde adquirieron el medicamento en los pacientes que acudieron a tres clínicas odontológicas privadas de lima en el año 2021

Variables	General		
	N.^o^	%
Recomendación		
Médico	36	9,3
Odontólogo	160	41,5
Farmacéutico	55	14,2
Técnico en farmacia	37	9,6
Familiar	46	11,9
Amistades	19	4,9
Por iniciativa propia	33	8,5
Motivo de automedicación		
Por indicación del técnico de farmacia	85	22,0
Porque es muy cara la consulta	63	16,3
Porque la clínica u hospital está muy lejos de su casa	64	16,6
Por hábito	92	23,8
Otros	82	21,2
Lugar donde adquirió el medicamento		
Farmacia	301	78,0
Botica	65	16,8
Bodega	6	1,6
Establecimiento de salud	14	3,6

En cuanto al tipo de medicamento más usado en la automedicación, se observó que los analgésicos representaron un 53,4% (n = 206) y en menor porcentaje los ansiolíticos, con un 1% (n = 1). Además, se identificó que la forma farmacéutica más utilizada fueron las pastillas, con el 96,9% (n = 374) (Tabla 4).

**Tabla 4:** Frecuencia y caracterización de automedicación según tipo y forma farmacéutica en los pacientes que acudieron a tres clínicas odontológicas particulares de Lima en el año 2021

Variables	General		
	N.^o^	%
Tipo de medicamento		
AINE	95	24,6
Antibióticos	60	15,5
Analgésicos	206	53,4
Ansiolíticos	4	1,0
Otros	21	5,4
Forma farmacéutica		
Pastillas	374	96,9
Jarabe	8	2,1
Suspensión	1	0,3
Intramuscular	3	0,8
Endovenosa	0	0

Según el medio de comunicación que influyó más en la automedicación en los pacientes, se encontró que la televisión representó el 73,1% (n = 282), lo que representó el mayor porcentaje respecto de los demás medios de comunicación (Tabla 5).

**Tabla 5:** Frecuencia y caracterización de automedicación según medio de comunicación en los pacientes que acudieron a tres clínicas odontológicas privadas de Lima en el año 2021

Variables	General		
	N.^o^	%
Medio de comunicación		
Televisión	282	73,1
Eslogan	30	7,8
Panel	29	7,5
Afiche	33	8,5
Radio	12	3,1
Total	386	100

**Figura 2:**
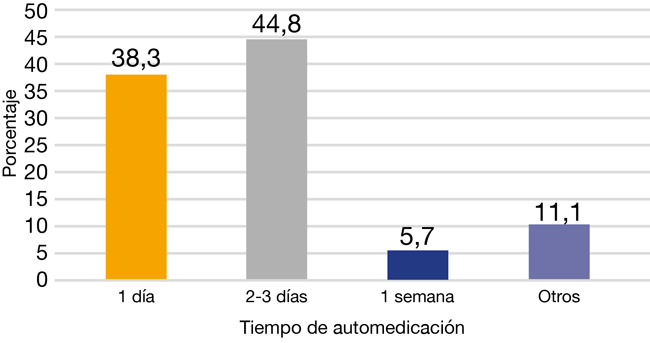
Frecuencia y caracterización de automedicación según tiempo en los pacientes que acudieron a tres clínicas odontológicas privadas de Lima en el año 2021

De acuerdo con la frecuencia y la caracterización de la automedicación según el tiempo, predominó la opción “2-3 días”, con un 44,8% (Figura 2).

## DISCUSIÓN

El presente estudio evidenció una asociación entre la automedicación y los pacientes que acuden a clínicas odontológicas de consulta privada, que cambia de acuerdo con la edad y no presenta mayor diferencia entre características como sexo, ocupación y grado de instrucción. Sin embargo, encontramos prevalencias elevadas de automedicación con respecto al tipo de dolencia, el motivo, la recomendación, el lugar de adquisición, el tiempo de medicación y los medios de comunicación que influenciaron a la práctica de la automedicación dentro de la muestra estudiada en Lima. 

Se halló que un 85,8% de los pacientes practicaban la automedicación en el sector odontológico. Esto coincide con los estudios realizados por Shrestha ^12^ en Katmandú (Nepal), con un 83,3%, y por AlQahtani ^1^ en Sharjah (Emiratos Árabes Unidos), con un 70,7%. En cambio, nuestro resultado fue mayor que el obtenido por Kassie ^13^ en el noreste de Etiopía, con un 35,9%. Esta prevalencia puede responder a los factores socioeconómicos, el tamaño de muestra y a que fue realizado en un distrito de la región. Por el contrario, el resultado del presente estudio fue menor al encontrado por Nogueira ^14^ en Perú, en el que el 100% de los participantes indicaron que se automedicaban. Esto pudo deberse a las facilidades de conseguir medicamentos sin ninguna receta y a la falta de concientización acerca de los efectos secundarios de consumir fármacos.

Se encontró que la mayor prevalencia de automedicación se daba en los pacientes entre 26 y 35 años, con un porcentaje del 35,5%. En los estudios realizado por KomalRaj ^15^ en Karnataka (India), Bhattarai ^16^ en Katmandú (Nepal) y Maquera ^17^ en Puno (Perú), se encontró que la prevalencia de automedicación era mayor en la mediana edad.

Respecto del género, se presentó con mayor frecuencia en el sexo femenino (50,3%), resultado que coincide con los estudios de Aldeeri ^18^ en Riad (Arabia Saudita), con un 69,44%, y de Shrestha ^12^, con un 80,6%. En cambio, un estudio realizado por KomalRaj ^15^ encontró un 61,7% para los participantes masculinos. Esto se puede deber a que la mujer tiene un umbral de resistencia más bajo del dolor y un mayor temor a los tratamientos dentales ^19^.

Respecto de la ocupación, se demostró que los pacientes profesionales fueron los que presentaron mayor frecuencia de automedicación, con un 40,2%. Este resultado puede responder al agitado estilo de vida en Lima, donde los pacientes profesionales no tienen tiempo para visitar al odontólogo. En cambio, un estudio realizado por Mavila ^20^ en Iquitos (Perú) demostró que el 37,2% tenían como ocupación la de comerciantes y en el estudio de Maquera ^17^ se halló que el 37,5% de los pacientes eran estudiantes.

En cuanto al grado de instrucción, se obtuvo que la mayor parte de pacientes con estudios universitarios concluidos (32,9%) se automedicaban. Esto coincide con los estudios realizados por AlQahtani ^1^, con un 46%, y Baptist ^21^, con un 63,33%. Esto se puede deber a que una persona con estudios tiene la competencia de interpretar las instrucciones de los medicamentos, tiende a tener confianza en sí misma y a tomar decisiones ^22^. En cambio, Nayyar ^23^, en su estudio realizado en Karnataka (India), halló que el 40% de los encuestados eran analfabetos. Este hallazgo se podría interpretar como que la ignorancia y la falta de educación podrían ser razones de la automedicación.

En este estudio se demostró que el 45,6% de los pacientes se automedicaban para aliviar el dolor dental, de igual manera que en los estudios realizados por Navabi ^24^ en Kerman (Irán), con el 44,3%; Nayyar ^25^ en Karnataka (India), con el 83,6%; y Olawuyi ^26^ en Lagos (Nigeria), con el 71%. Esto podría deberse a que el dolor principal se da en la zona bucal y, para la percepción de los pacientes, no es una enfermedad grave. En cambio, en un estudio realizado por Kalyan ^27^ se encontró que la halitosis era el problema más común, con un 11%, en Andhra Pradesh (India). Por su parte, en el caso de Simon ^28^, en Karnataka (India), fue la hinchazón en la cara, con un 17,5%.

En cuanto a las recomendaciones, el odontólogo fue quien recomendó el medicamento en el 41,5% de los casos. Escudero ^29^ en Córdoba (Argentina) obtuvo un 28%. Este resultado podría deberse a que los pacientes usaron la prescripción de su última cita dental y usaron el mismo medicamento para la automedicación. En cambio, en los estudios realizados por Jain ^30^ en Pradesh (India) y León ^31^ en Quito (Ecuador), fue el farmacéutico fue quiero asesoró la medicación (45% y 48,5%, respectivamente).

El motivo de la automedicación, según los resultados de este estudio, radica principalmente en que los pacientes consumen fármacos sin receta por hábito (23,8%). Esto podría atribuirse a que los pacientes han tenido una experiencia similar al dolor y consumieron el mismo fármaco que fue efectivo. En cambio, en los estudios realizados por AlQahtani ^1^ y Baptist ^21^, el principal motivo fue la falta de tiempo para visitar al odontólogo (37,6% y 65,33%, respectivamente).

En este estudio, el lugar predominante donde los pacientes adquirieron el medicamento sin prescripción médica fue la farmacia (78%). Este hallazgo guarda correspondencia con los estudios de KomalRaj ^15^ y Bhattarai ^16^, con porcentajes del 84% y el 73,5%, respectivamente. Esto se debió a que los participantes, a partir de sus conocimientos, pudieron adquirir el medicamento en un establecimiento de salud cercano.

El consumo autoadministrado de medicamentos puede provocar resistencia a los mismos. En este estudio se identificó a los analgésicos como el tipo de medicamento más consumido, con un 53,4%. Este patrón fue similar al de los estudios realizados por Simon ^28^, con el 42,5%, y Durrani ^32^, en Pakistán, con el 43,3%. Esto se debería a que, como el dolor dental es la razón principal para realizar la automedicación, recurren al uso de analgésicos, que están disponibles en las farmacias de manera libre. En cambio, en un estudio realizado por Stolbizer ^9^ en Buenos Aires (Argentina), la categoría más utilizada fueron los AINE, con un 61%, mientras que en el estudio de Baptist ^21^ el 53% de pacientes usó remedios caseros al menos en una ocasión para aliviar el dolor dental.

En el presente estudio, la forma de medicamento más usada fue la pastilla, con un 96,9%. Esto coincide con los estudios realizados por López ^33^ en Managua (Nicaragua), con un 68%, y los realizados por Escalona ^34^ en Barinas (Venezuela), con un 97,9%. Estos resultados pudieron deberse a la facilidad de acceso a las pastillas en los establecimientos de salud, sin requerimiento de una receta médica.

Los medios de comunicación son una gran influencia con respecto a los medicamentos que un paciente puede elegir para el alivio del dolor. En este estudio, el medio de comunicación que más influyó en los pacientes fue la televisión, con un 73,1%. Similar al estudio realizado por López ^33^, con un 73,9%, y Mayma ^35^ y Felipe ^36^, ambos en Perú, con un 58% y un 56%, respectivamente. Esto pudo deberse a que la televisión es el medio más popular y que está al alcance de la mayoría de los pacientes.

Finalmente, el tiempo de automedicación más usado por los pacientes en este estudio fue de 2-3 días, con un porcentaje del 44,8%. Este resultado es similar al de los estudios realizados por AlQahtani ^1^, con el 53%; Bhattarai ^16^, con el 59,6%, y KomalRaj ^15^, con el 60,6%. Estos hallazgos pueden responder a que el paciente, ante la manifestación del alivio del dolor, deja de usar el medicamento. En cambio, en el estudio de Durrani ^32^, el 35,8% de los pacientes se automedicaron 3 veces al año, y en el realizado por Gyawali ^37^ en el oeste de Nepal, el 29% indicó que se automedicaba más de 5 veces al año.

Una limitación del presente estudio durante su desarrollo es que, debido a la pandemia de COVID-19 y las restricciones asociadas, no se pudo efectuar una comparación entre los pacientes que acuden a establecimientos del sector público con respecto a los que acuden al sector privado. Por consiguiente, solamente se realizó la investigación en los pacientes que acudieron al sector privado. Se sugiere ampliar el estudio a otras poblaciones y realizar una investigación para reforzar el conocimiento sobre los efectos adversos que conlleva la automedicación

## CONCLUSIONES

Este estudió concluyó que la frecuencia de automedicación en esta población es alta y de mayor prevalencia en pacientes con estudios superiores. Se pudo observar también que el género femenino es más propenso a automedicarse. El principal motivo de automedicación fue el dolor dental y los pacientes usaron la prescripción de su última cita dental para aliviar dicho dolor.

La facilidad de conseguir medicamentos en farmacias sin receta prescrita por un especialista de la salud, la televisión como principal medio de comunicación y el simple hecho del hábito fueron también condiciones principales para la práctica de la automedicación.

Los analgésicos fueron el principal medicamento que usaron los pacientes para la automedicación y la forma farmacéutica por la que optaron mayoritariamente fueron las pastillas. Resulta de vital importancia educar y concientizar a los pacientes sobre las desventajas de consumir fármacos sin la guía de un profesional de la salud, pues en algunos casos podría habilitar la resistencia a los medicamentos, en especial en el grupo de los antibióticos.

## Fuente de financiamiento:

Autofinanciado
